# Health service security of patients with 8 certain rare diseases: evidence from China’s national system for health service utilization of patients with healthcare insurance

**DOI:** 10.1186/s13023-019-1165-7

**Published:** 2019-08-20

**Authors:** Rui Min, Xiaoyan Zhang, Pengqian Fang, Biyan Wang, He Wang

**Affiliations:** 1 Tongji Medical College, Huazhong University of Science& Technology, No.13 Hankong Road, Wuhan City, 430030 Hubei Province China; 2 College of Politics & Law and Public Administration, Hubei University, Wuhan, China; 3 School of Public Health and Management, Guangxi University of Chinese Medicine, Nanning, China; 4 Tongji Hospital, Huazhong University of Science and Technology, Wuhan, China

**Keywords:** Health consumption, Health security, Insurance coverage, Patients with rare disease

## Abstract

**Background:**

Rare diseases are one of the major challenges in the era of precision medicine and reflect the social security level of minority groups. This study aimed to investigate healthcare service utilization and health security of patients with rare diseases in China.

**Methods:**

From 29 provinces of Mainland China, 7,747 visits with eight common rare diseases who were linked to the national insurance database between 2014 and 2016 were selected as the study population, whose demographic and healthcare service information was collected from China’s national monitoring system for health service utilization of patients with healthcare insurance. Univariate analysis was performed to describe the basic statement of healthcare service, such as visit type, institution type, length of stay, healthcare insurance utilization, and the results of disease burden for different groups and its factors were analyzed by multivariate analysis.

**Results:**

Medical treatment from general tertiary hospitals was sought by 61.4% of the patients with rare diseases. Of the total treatment cost (TTC) of 40.18 million Chinese Yuan, 63.3% was paid by basic health insurance, and 54.2% of the medical cost resulted from medicine expenditure. Demography, geography and social-economic factors, security level, and health institution situation had an effect on the TTC. The correlations between these factors and TTC were different for outpatients and inpatients. Reimbursement rate had the highest effect on inpatients’ TTC. Basic insurance was effective for providing support for patients with rare diseases that involved high costs; however, the coverage was limited.

**Conclusions:**

Healthcare insurance is an effective safeguard for patients with rare diseases; however, affordable and accessible treatment is still lacking for such patients. There remains a need to further improve the diagnostic and treatment technology for rare diseases and expertise among doctors, as well as the security level of healthcare policies.

**Electronic supplementary material:**

The online version of this article (10.1186/s13023-019-1165-7) contains supplementary material, which is available to authorized users.

## Background

Rare diseases, also known as orphan diseases, refer to diseases that affect a small percentage of the population. With the deepening of research and reports on rare diseases, it is generally believed that even the rarity of each rare disease, the huge kinds and population with different rare disease sum up as a group making rare disease more common than public awareness [1, 2]. The World Health Organization (WHO) has estimated that more than 400 million people worldwide have one of the 7,000–8,000 diseases defined as rare [3], which means that one in 15 people is affected by a rare disease [4]. As a multi-ethnic country with 1.38 billion people, rare diseases are not considered rare in China. At least 90 million Chinese people have rare diseases. Although rare diseases have a low morbidity, some “common” rare diseases been, such as neuromuscular diseases, Fabry’s disease, Gaucher’s disease, phenylketonurias, hemophilia, myasthenia gravis, amyotrophic lateral sclerosis have a high public awareness in China [5, 6]. A previous study showed that more than 67.8% of doctors have encountered patients with rare diseases [7].

The prevention and treatment of rare diseases is a direct reflection of the level of social development and the development of healthcare system [8]. Rare diseases are one of the major challenges in the era of precision medicine because of the low incidence and prevalence, difficulty in diagnosis, lack of sufficient therapeutic methods, as well as their significant impact on the affected individuals, families, and the society [9, 10]. Most rare diseases are genetic and, thus, are present throughout the individual’s life, even if symptoms do not immediately appear. Many rare diseases appear early in life [11], while few patients of individual rare disease and complex biological mechanisms make the study of rare diseases further difficult. Due to the lack of a sufficiently large market, high costs hinder the development of orphan drugs for rare diseases [9, 12]. Effective and affordable treatment are the two most important and recent issues that need to be addressed [13].

Since 2012, the Chinese government has been making great efforts to reform the medical and healthcare system, and it was the same year that Present Xi and the Chinese government promoted “Healthy China” strategy, with the slogan “every citizen has the right to enjoy the basic healthcare service.” The New Healthcare reform and Healthy China Strategy aims to ensure stronger security for all Chinese citizens. With the rapid development of healthcare system, people’s level of health has greatly improved. A universal insurance coverage system, which includes three major medical insurance services, is available in China: the New Cooperative Medical Scheme (NCMS) for rural citizens, the Urban Employees’ Medical Insurance (UEBMI) and the Urban Residents Medical Insurance (URBMI) for citizens residing in cities [14]. UEBMI and URBMI are collectively known as Urban Basic Medical Insurance (UBMI), which covers 744 million citizens [15, 16]. With the development of society and economy, it gives a great stimulate to the release of health demand, which lead to more cost in health, and high risk and financial burden for the nation. Pay by diagnostic related groups (DRGs) had been introduced to the insurance payment reform in the late of 1990’s in China. It began with the usage of settlement by disease type and value (point method). Since 2011, Pay-by-DRGs had been more and more popular, and many regions had taken it as an effective reform try. Nowadays, use of diagnostic related groups-prospective payment system, DRGs-PPS, has been an observed tendency in the payment reform of health care insurance, to cut down the medical expenditure and risk control [17, 18].

Notably, the focus of Chinese healthcare system is the diagnosis and treatment of diseases with large number of patients; however, according to the treatment reported by the National Health Commission of China (NHC), patients with rare diseases have very few options of treatment and diagnosis in China. Compared with the national list of 121 rare disease and 1010 clinical pathways, there were only 17 clinical pathways for rare diseases, and the first 21 orphan drugs had been included in the national healthcare insurance list till to 2019 [19]. It is difficult to verify the coverage of the rare disease due to the scarcity of appropriate medicines and treatment [8]. Furthermore, without specific policies in insurance for rare diseases, patients may experience high financial burden which may lead to Catastrophic health expenditure (CHE) [20–22]. Although the series of research studies, clinical diagnosis, and treatment of rare diseases in China have seen positive results in the recent years, with the development of the society, progress of technology, the rare disease population are expanding with the growing number of diagnosed and treatable rare diseases, which could be detrimental to the economy and social stability, and lead to serious public health and social problems [9, 23–25]. Understanding the utilization of health insurance among patients with rare diseases is important for the development of a national and regional healthcare system [8, 26]. Limited studies of the health service utility for rare diseases patients are reported given the unclear diagnoses, complex treatment, and minority group of rare disease [5, 27–29]. Based on the utilization information of healthcare service of patient with rare diseases between 2014 and 2016, this study aimed to investigate healthcare service utilization and the basic situation of healthcare security of patients with rare diseases in China. This study was the first national report of the healthcare expenditure and healthcare insurance utilization of patients with rare diseases in Mainland China.

### Patients and methods

#### Database introduction and data collection

In 2008, the Chinese government established a routine reporting system for health service utilization of patients with UBMI, which included available data from sample cities (all cities in the database) in all 31 provinces of Mainland China. Based on the proposal of China State Statistical Bureau, all provinces were divided into three areas for statistical analysis according to the economic development and geographical position when statistical analysis is conducted. Data from the special administrative region and Taiwan Province were excluded from this study. The eastern area refers to developed areas, including 11 provinces or municipalities. The central area refers to developing areas, including eight provinces. The western area refers to underdeveloped areas, including 11 provinces or autonomous. The basic status of different areas of China was in Additional file 1: Appendix I.

This database, operated by the China Healthcare Insurance Research Association (CHIRA), covers all the healthcare utility data in hospitals from all the 31 provinces in Mainland China [30, 31]. The basic information of the database is shown in Table 1.

**Table 1:** Basic information for the national database

	Statistic Year		
	2014	2015	2016
Total number of insurance utility			
national level (people × times × 1,000,000)	571	597	666
sample (people × times × 1,000,000)	49.57	41.79	37.26
sample cities	77	77	82
direct-controlled municipality	4	4	4
provincial city^a^	31	31	31
prefecture-level city	42	42	47
inpatient (people × 1,000,000)	1.36	1.13	1.47
outpatient (people × 1,000,000)	4.86	4.81	6.53
outpatient (people × time × 1,000,000)	40.67	37.22	35.78

Note: a) there are 34 provinces in China, data of Hong Kong, Macao, and Taiwan were not included in the database as different insurance type

We extracted data from the 2014–2016 from the national database. The data contained the basic demographic information and medical service utility information, which included information about the disease diagnosis, medical expenses, and insurance coverage. It should be noted that a statistical year is the year after the civil year, which means that the data in statistical years 2014–2016 were recorded between civil years 2013 and 2015.

#### Data statement

Data collected from yearbook may be available in the report listed in References [14]. And all patients’ information and healthcare cost were collected from CHIRA’s insurance database mentioned previously. According to the statistic law of People’s Republic of China and the confidentiality agreement between research team and CHIRA, the research team must keep the security of the initial data. Data may be available from the corresponding author after any reasonable request or get from CHIRA.

#### Procedure and participant

As mentioned previously, although a low morbidity makes rare disease rare and difficult to identify, there are some rare diseases with high media exposure and public awareness, which can be referred to “common” rare disease. The selection of rare diseases of the present study was processed during later in 2016 to earlier in 2017. According to the low morbidity, hard to diagnose and rare of medicine and treatment, it was difficult to figure out the appropriate rare diseases in the national insurance utility system in the absence of a national list of rare diseases. The research team first started with literature review to choose rare diseases that had high frequency in both clinical and public reports, then a discussion group had 3 professors major in rare disease and healthcare security helped in compiling a list of 20 certain rare diseases, with a high morbidity rate and those familiar to Chinese public [32–34]. Then ten rare diseases were selected randomly from the 20 options. We submitted the application of health service utilization data of the certain rare diseases to CHIRA. After the data feedback, two rare diseases with ultra-low incident rate (patient less than 10 in 3 years) were excluded from the database. These 8 study rare diseases included acute promyelocytic leukemia (APL), growth hormone deficiency (GHD), haemophilia, motor neuron disease (MND), mucopolysaccharidosis (MPS), multiple sclerosis (MS), myasthenia gravis (MG), phenylketonuria (PKU). (The selection diseases and procedure are provided in Additional file 1: Appendix II).

The study population consisted of male and female patients in China diagnosed with rare diseases, who received treatment from medical institutions. The inclusion criteria were as follows: Chinese residents, who were covered by the basic health insurance, diagnosed of one of the 8 rare diseases with specific diagnostic criteria, well-developed treatment protocol, or curable or treatable rare diseases with a high survival rate [29, 35–45]. Basic information of patients with the 8 disease are shown in Table 2). Rare disease was the main diagnosis shown in the records from CHIRA, and International Classification of Diseases (ICD) code was used to filter and match patients with the certain rare diseases. Patients with an unclear or no diagnosis of a rare disease were excluded from this study. A unique identification code for visit records was used, as the personal information removed for the privacy protection.

**Table 2:** Basic condition for the 8 rare diseases

	Obs. [n(1/10^6^)]	Medical cost					
		TTC (¥. RMB)		INSURAPAY (¥. RMB)		R_*reimbursement*_ (%)	
	sample(incident rate) ^a^	mean	medium	mean	medium	mean	medium
Total	7,747(65.8)	5180.12	533.44	3279.93	334.28	67.1	72.1
Disease type							
Acute promyelocytic leukemia, APL	660(5.6)	12167.46	7322.63	7635.91	4616.91	63.2	67.5
Growth hormone deficiency, GHD	94(0.8)	2419.08	1312.88	1352.71	965.82	54.7	60.6
Hemophilia	1,517(12.9)	4957.53	1659.20	3408.02	876.16	64.8	71.0
Motor neuron disease, MND	958(8.1)	10619.24	571.64	5744.06	350.09	71.3	78.4
Mucopolysaccharidosis, MPS	20(0.2)	6858.53	6053.39	4635.00	4526.71	57.8	65.7
Multiple sclerosis, MS	775(6.6)	5042.85	547.00	3449.75	383.09	69.5	72.1
Myasthenia gravis, MG	3,683(31.3)	2744.99	312.29	1842.70	208.06	67.4	72.7
Phenylketonuria, PKU	40(0.3)	587.55	20.00	425.44	20.00	69.1	65.5

Note: a) incident rate was the percentage of health service records with main diagnosis of rare disease in all the health service records in the database

#### Definitions of health security indicators

The total treatment cost (TTC) is defined as the total cost of medical care for a patient in a year. In this study, TTC represents the director cost for the diagnoses and treatment for rare disease, including costs of drugs, examinations, consultations, treatments, inpatient stays, and other direct healthcare services. Indirect costs, like the costs of transportation, special diets, and company of family, and income lost due to illness, were not considered in this study.

There were differences in the target population, enrollment type, premiums, and reimbursement rates among the three basic healthcare insurance [46] (Additional file 1: Appendix III). The health security system has been, and still is, operated at the provincial level; this provides the flexibility to tailor the system according to the regional socio-demographic and fiscal needs. It has also led to an effective reimbursement coverage varied across provinces [46, 47]. Generally, TTC could be divided into two parts according to the insurance payment, the total reimbursement which paid by the insurance, and the out-of-pocket costs (OOP) paid by person:

$$ \mathrm{TTC}=\mathrm{total}\ \mathrm{reimbursement}+\mathrm{OOP} $$

However, there is a fixed amount within the total reimbursement, which the health insurer requires a patient to pay for a medical service. Therefore,

$$ \mathrm{TTC}=\mathrm{effective}\ \mathrm{reimbursement}\ \mathrm{coverage}+\mathrm{co}-\mathrm{payment}+\mathrm{self}-\mathrm{payment} $$

$$ \mathrm{OOP}=\mathrm{TTC}-\mathrm{effective}\ \mathrm{reimbursement}\ \mathrm{coverage} $$

$$ \mathrm{Also},\mathrm{OOP}=\mathrm{co}-\mathrm{payment}+\mathrm{self}-\mathrm{payment} $$

The share of medical expenses reimbursed by the insurance represents the medical guarantee. The Chinese health insurance policy categorizes TTC into two components—expenses within and beyond the payment scope of basic medical insurance [46]. All the health security indicators mentioned above could be derived directly from the national database.

Another important indicator for the evaluation of the security level of health insurance is the reimbursement rate. Generally, there are two healthcare insurance reimbursement rates in China—the medical guarantee stipulated in the current policy and the actual medical guarantee provided. For the convenience of understanding and comparison, we only used the actual medical reimbursement rate (*R*_*reimbursement rate*_) in this study, which is computed as follows:

$$ {R}_{reimbursement\ rate}=\mathrm{aggregate}\ \mathrm{in}\mathrm{surance}\ \mathrm{benefit}\ \mathrm{received}\ \mathrm{in}\ \mathrm{a}\ \mathrm{single}\ \mathrm{policy}\ \mathrm{year}/\mathrm{TTC}\ast 100\% $$

CHE refers to health expenditure that threatens a household’s capacity ability to maintain a basic standard of living, and could be regarded as a key monitoring indicator of poverty caused by the illness as well as the security level of the healthcare insurance. According to the WHO’s definition, when the household medical expenditure exceeds 40% of the household consumption, it is considered a CHE [48]. This circumstance was equal in China that, when the individual burden of medical expense reaches the per capita annual disposable income of urban residents, or arrives at the annual per capita incomes of farmers, the patient or his family will suffer from CHE [49, 50]. The provincial annual per capita disposable income from the national statistic year book was added to analysis the CHE of rare diseases.

#### Statistical analysis

First, the study used descriptive analysis to describe the basic condition of the sample patients, including their demographic data, disease diagnosis, medical expenses, and insurance coverage. Continuous variables were expressed as mean ± SD, while categorical data were presented as proportions. *T*-test was performed for normally distributed continuous data or Chi-square test for non-normally distributed data. ANOVA was performed for categorical data. A two-sided *P* < 0.05 was considered statistically significant. We also estimated the prevalence and medical cost of the eight rare diseases. Second, correlation analysis was performed to examine the relationship among different factors. Ordinary Least Squares (OLS) Regressions and Quantile Regression was used to identify the financial burden among different groups and its influencing factors, TTC was split by 6 points (percentiles 0.1, 0.25, 0.5, 0.75,0.9 and 0.98) to compare changes of influencing factors in different consumption groups. Among the consumption groups, patients with TTC on the bottom 10% was named 10% low-cost group, and patients whose TTC on the above 90% was the 90% high-cost group.

The influencing factors were defined a priori by all possible combinations of ten selected characteristics: age (0–5, 6–19, 20–29, 30–39,40–49, 50–59, 60–69, 70–79, and over 80 years), sex (male and female), geographical region (Western, Central, and Eastern China), city level (a four-level classification defined by State Council of China according to the population and the situation of the social and economic development of the city; level 1 indicates “most developed city” and level 4 indicates “least developed city”), type of health institution (class III, II, I hospital), specialization type (general hospital and specialized hospital), insurance type (URBMI and UEBMI), service type (inpatient and outpatient), insurance payment type (Pay-by-DRGs; or traditional payment method), and length of stay (LOS). Disease type was not included as the influencing factors in the regression analysis, because in quantile regression, the more classifications of categorical independent variables, the weaker the effect of the explanation for the dependent variable. All 10 characteristics along with the disease type, security indicators (TTC, total reimbursement, OOP, and co-payment) could be extracted from the database. Data of CHE was analysed with the provincial annual per capita disposable income from the national statistic year book. All statistical analyses were conducted using STATA 15.0.

## Results

### Basic conditions of patients with rare diseases

**Fig. 1:**
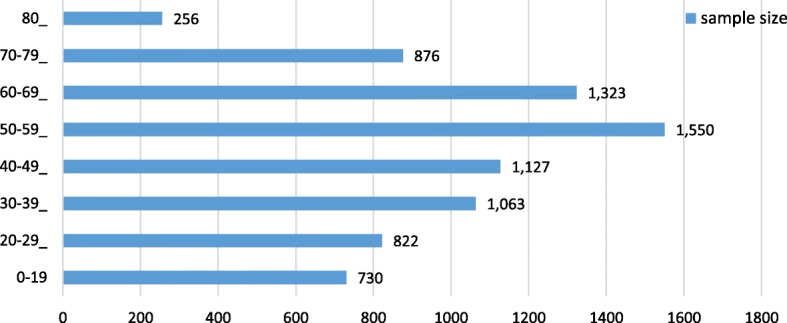
Age distribution of sample population

**Fig. 2:**
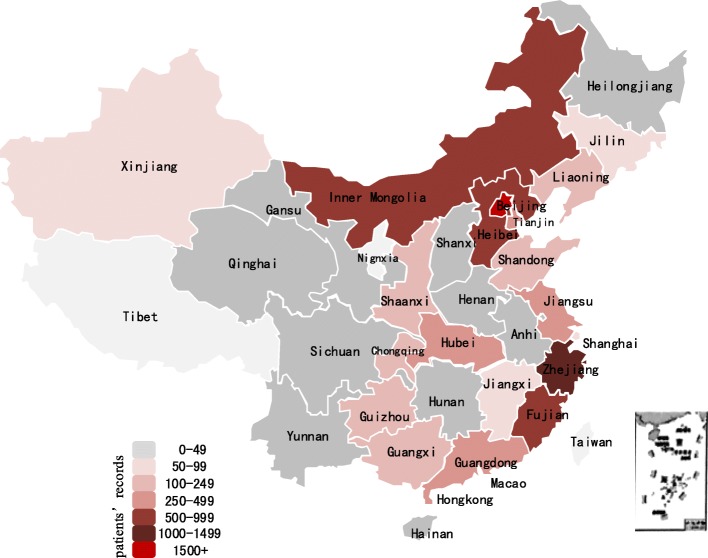
Map of basic insurance records for patients with the 8 selected rare diseases (2014–2016) in Mainland China. Note: there is no basic insurance record for patients with the 8 selected rare diseases in Tibet, Ningxia, Taiwan, Hong Kong and Macao during the study period

This study included 7,747 records from 29 provinces in Mainland China, excluding Tibet and Ningxia (Figs. 1 and 2). The average TTC was Chinese Yuan ¥5,180 (UK dollar £580), in which the basic insurance paid for ¥3,280 (£367). Patients with APL, MND and MPS had higher expenses than the average level, patients with APL had the highest average TTC, while patients with PKU hold the lowest average TTC. The reimbursement rates for different rare diseases was basically at the same level. GHD had the lowest reimbursement rate (*R*_reimbursement rate ‐ GHD_ = 60.6%), while MND had the highest reimbursement rate (*R*_reimbursement rate ‐ MND_ = 78.4%) (Table 2).

The average age of all the health service utilization records was 47.76 years, and 45.3% of them were by females. Overall, 73.8% of the records were from Eastern China, and 31.0% were from a level 3 city. The UEBMI covered 82.2% of the present study’s sample (Tables 3 and 4).

**Table 3:** Basic information for healthcare service of sample during 2014 to 2016 (categorical variable)

Variable [*n*(%)]	Total	By year		
		2014	2015	2016
Obs. (n)	7,747	2,553	2,780	2,414
Gender	7,747			
Male	4,236(54.7%)	1,339(52.4%)	1,595(57.4%)	1,302(53.9%)
Female	3,511(45.3%)	1,214(47.6%)	1,185(42.6%)	1,112(46.1%)
PAYBY DRGs	7,747			
Using DRGs	3031(39.1%)	804(31.5%)	1272(45.8%)	955(39.6%)
Insurance type	7,747			
UEBMI	6,369(82.2%)	2,104(82.4%)	2,400(86.3%)	1,865(77.3%)
URBMI	1,378(17.8)	449(17.6%)	380(13.7%)	549(22.7%)
AREA	7,747			
Eastern area	5,714(73.8%)	2,075 (81.3%)	1,829(65.8%)	1,810(75.0%)
Central area	614(7.9%)	165(6.5%)	244(8.8%)	205(8.5%)
Western area	1,419(18.3%)	313(12.3%)	707(25.4%)	399(16.5%)
City-level	7,747			
Class 1 city	2,064(26.6%)	724(28.4%)	756(27.2%)	584(24.2%)
Class 2 city	2,005(25.9%)	412(16.1%)	803(28.9%)	790(32.7%)
Class 3 city	2,403(31.0%)	1,327(52.0%)	496(17.8%)	580(24.0%)
Class 4 city	1,275(16.5%)	90(3.5%)	725(26.1%)	460(19.1%)
Visit type	7,747			
Inpatient visit	2,305(29.8%)	1,021(40.0%)	534(19.2%)	750(31.1%)
Outpatient visit	5,442(70.2%)	1,532(60.0%)	2,246(80.8%)	1,664(68.9%)
Institution type	7,747			
Class III hospital	5,626(72.6%)	2,065(80.9%)	1,779(64.0%)	1,778(73.6%)
Class II hospital	805(10.4%)	244(9.6%)	265(9.5%)	296(12.3%)
Class I hospital	1,322(17.1%)	244(9.6%)	736(26.5)	340(14.1%)
Hospital for special	7,747			
General hospital	5,657(73.0%)	2,034(79.7%)	1,780(64.0%)	1,843(76.3%)
Special hospital	1,648(21.3%)	4,66(18.3%)	767(27.6%)	415(17.2%)
Clinic & Community	442(5.7%)	53(2.1%)	233(8.4%)	156(6.5%)

**Table 4:** Basic information for healthcare service of sample during 2014 to 2016 (continuous variable)

	Total	By year		
		2014	2015	2016
Obs. (n)	7,747	2,553	2,780	2,414
Variable [mean]				
Age (year)	47.76	46.59	47.41	49.39
LOS^a^(day)	13.46	15.17	14.59	10.34
TTC (¥)	5,180	6,548	3,752	5,378
Medicine cost(¥)	2,811	3,529	2,059	2,919
Treatment cost(¥)	2,228	2,781	1,463	2,525
Pay-by-DRGs cost(¥)	1,521	649	1,798	2,198
Pay by insurance(¥)	3,280	4,476	2,444	2,977
OOP(¥)	1753	1,973.	1,016	2,368
COPAY(¥)	827	1,084	638	774
SELFPAY(¥)	1,073	988	670	1,626

Note: ^a^ inpatients obs = 2305

¥represent Chinese yuan

**Fig. 3:**
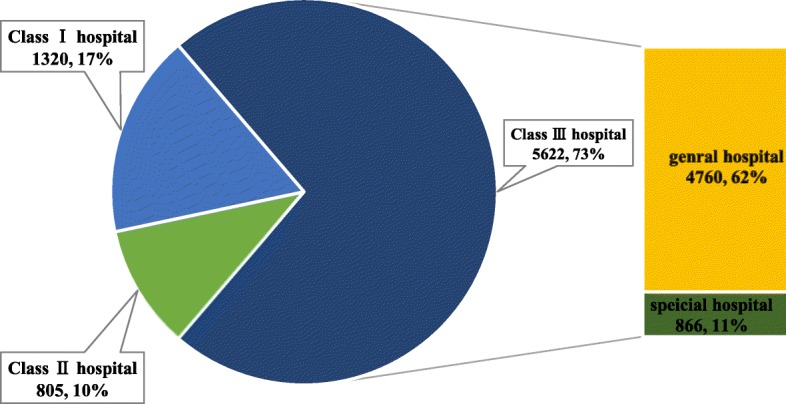
Pie chart of patient records in different types of medical institutions

Generally, 5,657 cases were reported in general hospitals while 72.6% sought treatment from a tertiary level /Class III hospital (Fig. 3). In particularly, 2,305 records were inpatients with an average LOS of 13.5 days, which had dropped from 15.17 days in 2014 to 10.34 days in 2016 (Table 4). Meanwhile, 66.3% of the outpatient cases received treatment in general hospitals. Compared to inpatients, 17.4% more outpatients chose specialized hospitals, while community hospitals had 5.3% more outpatient visits than inpatient visits.

Three thousand thirty-one records used pay-by-DRGs method, which covered ¥1,521 (£170) on average. The coverage rate of pay-by-DRGs was 31.5% in 2014, which had increased up to 39.6% in 2016, while the average payment increased by ¥1,549 (£173) during the same period.

### Medical cost for different visit type patients with rare diseases

For the inpatient service, average TTC per visit was ¥15,724 (£1,758) which was 2.0 times higher than the national average medical cost (Additional file 1: Appendix IV). Among the total cost, 54.3% cost was paid for medicine, and only 26.9% was paid by DRGs. Meanwhile, the TTC of 10.8% of the inpatients was higher than the per capita disposable income at the provincial level, which indicated that this group may suffer from CHE. A total of 2,273 visitors were covered by insurance, and the *R*_*reimbursement rate*_ was 70% (Tables 5, 6, 7 and 8).

**Table 5:** TTC of sample during 2014 to 2016

	Total	By year		
		2014	2015	2016
TTC (¥) on average	5,180	6,548	3,752	5,378
Inpatient TTC	15,723.65	14,893.68	16,744.09	16,126.97
Outpatient TTC	714.33	985.83	663.50	532.97

**Table 6:** Details of medical cost for inpatients and outpatients with rare diseases during 2014 to 2016

Visit type		Medical cost (¥. RMB)						
		TTC	INSURAPAY	Co-pay	OOP	Medicine	Treatment	Pay-by-DRGs
Inpatient	Total	36,243,016.87	22,571,132.30	5,708,073.69	12,499,232.76	18,409,590.65	16,793,682.38	8,802,491.73
	Average	15,723.65	9,792.25	2,476.39	5,422.66	7,986.81	7,285.76	4,169.82
	S.E.	972.58	388.91	112.37	778.43	397.29	586.28	973.75
	Medium	8,194.98	5,671.46	1,500.00	2,061.00	3,948.39	3,391.90	0.00
Outpatient	Total	3,887,375.58	2,838,454.75	700,178.87	1,080,040.02	3,368,924.60	469,458.00	1,998,834.16
	Average	714.33	521.58	128.66	198.46	619.06	86.27	400.57
	S.E.	26.03	22.68	5.71	9.28	25.54	4.30	15.98
	Medium	241.24	145.90	25.09	33.51	142.90	2.00	29.48
Total	Total	40,130,392.45	25,409,587.04	6,408,252.56	13,579,272.78	21,778,515.25	17,263,140.38	10,801,325.89
	Average	5,180.12	3,279.93	827.19	1,752.84	2,811.22	2,228.36	1,521.10
	S.E.	300.21	126.33	35.81	233.25	125.52	178.40	290.37
	Medium	533.44	334.28	74.43	108.72	351.28	18.53	0.00

**Table 7:** Payment structure of TTC for patients with rare diseases during 2014 to 2016 (by year)

	Total(mean& medium)	By year		
		2014	2015	2016
Reimbursement rate(%)	67.1(72.1)	72.9(76.0)	67.6(71.3)	60.3(70.0)
Proportion of OOP^a^	32.9(27.9)	27.1(24.0)	32.4(28.7)	39.7(30.0)
Copay rate(%)	22.2(14.9)	16.6(10.1)	22.6(15.8)	27.7(15.0)
Selfpay rate(%)	10.7(0.9)	10.5(2.7)	9.8(0.0)	12.0(0.6)

*a*: the national annual report data showed that proportion of OOP in health pay was 33.9%m 32.0 and 29.3% respectively

**Table 8:** Payment structure of TTC for patients with rare diseases during 2014 to 2016 (by visit type)

		R _*reimbursement rate*_ (%)	Copay rate(%)	OOP rate(%)
Visit type (mean& medium)	inpatient	67.0(70.0)	21.5(18.7)	33.0(30.0)
	outpatient	67.1(75.0)	22.5(12.0)	32.9(25.0)

For the outpatient service, average TTC per visit was ¥714.3 (£79.9), 3.2 times higher than the national average cost, and the insurance paid ¥521.23 (*R*_*reimbursement rate*_ = 72.98%) (Tables 5, 6 and 7). Despite being covered by basic insurance, there were still 648 (11.9%) cases reported 100% OOP during their visit time.

### Coverage level for the basic insurance of different subgroup patients

With an in-depth evaluation of TTC of patients with rare disease, the results of the correlation analysis showed a significant correlation between the TTC and geographical and economical distribution (area and city level), demographical factors (gender and age) at the1% significance level. While, OOP had no significant difference among different areas. Moreover, the TTC was influenced by institution where the patient chose to get service *(P < 0.01)*. Meanwhile, different health care payment methods also affected the TTC. The results showed a positive correlation between TTC and pay-by-DRGs—a method of the insurance payment reform—during the study period (average TTC _*pay-by-DRGs*_ = ¥6056.7, *P < 0.01*) (Table 9).

**Table 9:** TTC, OOP and reimbursement rate of different subgroups

Variable		TTC(¥. RMB)		OOP(¥. RMB)		R _*reimbursement rate*_ (%)	
		mean	*p*	mean	*p*	mean	*p*
Area	Eastern area	4,211.6	< 0.01	1,278.2	0.521	65.3%	< 0.01
	Central area	13,114.9		7,035.7		65.0%	
	Western area	5,646.6		1,378.4		75.3%	
City Level	1	1,880.0	< 0.01	473.8	< 0.01	63.2%	< 0.01
	2	4,492.1		1,219.7		65.7%	
	3	8,146.2		2,571.1		67.9%	
	4	6,014.1		3,119.7		74.1%	
Insurance Type	UEBMI	6,082.1	< 0.01	2,189.3	< 0.01	64.6%	< 0.01
	URBMI	5,271.0		1,322.5		67.4%	
Gender	Male	1,283.1	< 0.01	156.5	< 0.01	77.5%	0.046
	Female	5,863.1		2,081.6		67.7%	
Age	0–5	4,356.2	< 0.01	1,356.2	< 0.01	66.4%	< 0.01
	6–19	2,775.6		628.6		70.5%	
	20–29	4,147.6		1,774.8		57.4%	
	30–39	6,272.7		2,123.7		61.2%	
	40–49	4,663.8		1,236.2		68.3%	
	50–59	5,506.1		1,907.5		70.0%	
	60–69	4,622.4		1,328.5		68.3%	
	70–79	6,901.4		3,248.8		68.1%	
	80-	3,928.7		834.1		69.7%	
Visit Type	Inpatient	5,090.4	< 0.01	783.6	< 0.01	64.7%	< 0.01
	Outpatient	6,082.1		2,189.3		64.6%	
Institution Type	Class III hospital	5,271.0	< 0.01	1,322.5	< 0.01	67.4%	< 0.01
	Class II hospital	1,283.1		156.5		77.5%	
	Class I hospital	5,984.9		1,952.1		65.1%	
Hospital For Special	General hospital	3,644.6	< 0.01	1,505.7	< 0.01	72.6%	< 0.01
	Special hospital	605.0		124.3		71.8%	
	Clinic & community	3,816.3		1,490.3		68.7%	
Pay-by-DRGs	Yes	6,056.7	< 0.01	1,921.6	< 0.01	66.0%	< 0.01
	No	4,211.6		1,278.2		65.3%	

*Note: Spearman Two-Tail No-Sig was used to the nonparametric tests*

### Regression analysis between TTC and different influencing factors

In terms of comparison, the OLS model suggests that the age, LOS, *R*_*reimbursement rate*_, OOP, gender, city level, visit type, pay-by-DRGs, patients from Central Area, and treatment of Class I hospital, or first visit in clinics had a statistically significant effect on the TTC at the 1% significance level (Tables 10, 11 and 12). Age, LOS, *R*_*reimbursement rate*_, OOP, areas, institution type, special hospital and pay-by-DRGs showed significant effect on the TTC for inpatients at the 1% level, while for outpatients, all the factors showed significant effect on the TTC.

**Table 10:** Results of ordinary least squares (OLS) and quantile regression

Ln TTC	OLS	QR_10	QR_25	QR_50	QR_75	QR_90	QR_98
AGE	− 0.004^c^	0.006^a^	0.006^c^	−0.003^b^	− 0.005^c^	− 0.010^c^	−0.011^c^
	(0.001)	(0.001)	(0.001)	(0.001)	(0.001)	(0.001)	(0.002)
LOS	.031^c^	0.037^c^	0.037^c^	0.038^c^	0.036^c^	0.027^c^	0.025^c^
	(0.002)	(0.003)	(0.003)	(0.003)	(0.002)	(0.003)	(0.003)
R _*reimbursement rate*_	0.427^c^	0.528^c^	0.528^c^	0.541^c^	0.669^c^	0.881^c^	1.233^c^
	(0.060)	(0.087)	(0.087)	(0.064)	(0.061)	(0.080)	(0.099)
OOP	0.000007^c^	0.00001^c^	0.00001^c^	0.00006^c^	0.00009^c^	0.0002^c^	0.0002^c^
	(0.000)	(0.000)	(0.000)	(0.000)	(0.000)	(0.000)	(0.000)
GENDER							
Male	0	0	0	0	0	0	0
Female	−0.248^c^	−0.0985^b^	−0.0985^b^	−0.265^c^	− 0.239^c^	− 0.328^c^	− 0.368^c^
	(0.034)	(0.049)	(0.049)	(0.036)	(0.035)	(0.045)	(0.056)
AREA							
Eastern Area	0	0	0	0	0	0	0
Central Area	0.312^c^	0.0154	0.0154	0.0278	0.139^b^	0.260^c^	0.140
	(0.069)	(0.099)	(0.099)	(0.073)	(0.070)	(0.091)	(0.114)
Western Area	−0.092^a^	− 0.182^b^	−0.182^b^	−0.133^b^	− 0.086	0.060	− 0.010
	(0.053)	(0.076)	(0.076)	(0.056)	(0.0541)	(0.070)	(0.088)
CITY LEVEL							
Level 1	0	0	0	0	0	0	0
Level 2	−0.335^c^	−0.317^c^	− 0.317^c^	− 0.131^b^	−0.120^b^	− 0.158^b^	−0.361^c^
	(0.050)	(0.072)	(0.072)	(0.053)	(0.051)	(0.066)	(0.082)
Level 3	−0.623^c^	− 0.548^c^	−0.548^c^	− 0.288^c^	−0.295^c^	− 0.460^c^	−0.570^c^
	(0.060)	(0.086)	(0.086)	(0.063)	(0.061)	(0.080)	(0.099)
Level 4	−0.794^c^	− 0.661^c^	−0.661^c^	− 0.625^c^	−0.561^c^	− 0.825^c^	−0.875^c^
	(0.075)	(0.108)	(0.108)	(0.080)	(0.077)	(0.100)	(0.124)
VISIT TYPE							
Inpatient	0	0	0	0	0	0	0
Outpatient	−3.611^c^	−4.067^c^	−4.067^c^	−3.222^c^	−2.441^c^	−1.935^c^	−1.597^c^
	(0.050)	(0.072)	(0.072)	(0.053)	(0.051)	(0.067)	(0.083)
INSURANCE TYPE							
UEMBI	0	0	0	0	0	0	0
URMBI	−0.0443	− 0.118	− 0.118	− 0.0305	0.117^b^	0.0607	0.0736
	(0.051)	(0.073)	(0.073)	(0.054)	(0.052)	(0.068)	(0.084)
INSTITUTION TYPE							
Class III hospital	0	0	0	0	0	0	0
Class II hospital	−0.0593	− 0.00363	− 0.00363	0.00649	− 0.117^b^	− 0.138^a^	− 0.175^a^
	(0.051)	(0.073)	(0.073)	(0.054)	(0.052)	(0.068)	(0.084)
Class I hospital	0.604^c^	0.723^c^	0.723^c^	0.486^c^	0.168^c^	0.635^c^	0.861^c^
	(0.0595)	(0.0859)	(0.0859)	(0.0631)	(0.0609)	(0.0790)	(0.0986)
Hospital for special							
General hospital	0	0	0	0	0	0	0
Special hospital	0.0876^a^	0.123^a^	0.123^a^	0.112^b^	−0.0400	− 0.0733	− 0.195^c^
	(0.056)	(0.080)	(0.080)	(0.059)	(0.057)	(0.074)	(0.092)
Clinic & Community	−1.200^c^	−1.546^c^	−1.546^c^	−0.842^c^	− 0.754^c^	−1.370^c^	− 1.884^c^
	(0.060)	(0.086)	(0.086)	(0.063)	(0.061)	(0.079)	(0.099)
Pay-by-DRGs							
Yes	0	0	0	0	0	0	0
No	−0.307^c^	− 0.314^c^	− 0.314^c^	− 0.477^c^	− 0.382^c^	−0.238^c^	− 0.181^b^
	(0.047)	(0.068)	(0.068)	(0.050)	(0.048)	(0.063)	(0.079)
cons	9.398^c^	8.282^c^	8.282^c^	8.967^c^	9.112^c^	9.385^c^	9.651^c^
	(0.086)	(0.125)	(0.125)	(0.092)	(0.088)	(0.115)	(0.143)
*No.Obs*	7747	7747	7747	7747	7747	7747	7747
*R*^2^	0.617						
Pseudo *R*^2^		0.351	0.389	0.447	0.490	0.466	0.476

Note: as TTC was abnormal distribution, ln TTC was used to make the variable normalized

Standard errors in parentheses

^a^ Significant at the 90% level., ^b^ Significant at the 95% level, ^c^ Significant at the 99% level

**Table 11:** Results of ordinary least squares (OLS) and quantile regression for inpatients

Ln TTC	OLS	QR_10	QR_25	QR_50	QR_75	QR_90	QR_98
AGE	0.004^c^	0.006^c^	0.005^c^	0.004^c^	0.001	0.0003	− 0.0003
	(0.001)	(0.002)	(0.001)	(0.001)	(0.001)	(0.001)	(0.002)
LOS	0.027^c^	0.030^c^	0.036^c^	0.032^c^	0.025^c^	0.021^c^	0.020^c^
	(0.001)	(0.002)	(0.001)	(0.001)	(0.001)	(0.001)	(0.002)
R _*reimbursement rate*_	0.450^c^	1.061^c^	0.903^c^	0.794^c^	1.143^c^	1.384^c^	1.665^c^
	(0.100)	(0.163)	(0.119)	(0.101)	(0.100)	(0.116)	(0.198)
OOP	0.000006^c^	0.000003^c^	0.000009^c^	0.00006^c^	0.00008^c^	0.0001^c^	0.0001^c^
	(0.000)	(0.000)	(0.000)	(0.000)	(0.000)	(0.000)	(0.000)
GENDER							
Male	0	0	0	0	0	0	0
Female	0.0411	0.0256	0.0411	−0.0129	−0.0328	−0.123^c^	−0.265^c^
	(0.037)	(0.060)	(0.044)	(0.037)	(0.036)	(0.043)	(0.073)
AREA							
Eastern Area	0	0	0	0	0	0	0
Central Area	−0.170^c^	0.0872	− 0.100^a^	− 0.127^b^	− 0.179^c^	− 0.157^c^	−0.212^b^
	(0.051)	(0.083)	(0.061)	(0.052)	(0.051)	(0.059)	(0.101)
Western Area	−0.200^c^	− 0.0407	− 0.218^c^	− 0.160^c^	−0.0795^a^	0.00839	0.177^b^
	(0.043)	(0.070)	(0.051)	(0.044)	(0.043)	(0.050)	(0.085)
CITY LEVEL							
Level 1	0	0	0	0	0	0	0
Level 2	0.169^a^	0.128	0.114	0.0195	0.0439	0.154	0.21
	(0.090)	(0.147)	(0.107)	(0.091)	(0.089)	(0.105)	(0.178)
Level 3	0.087	0.0835	0.0834	−0.063	−0.0633	−0.0102	−0.116
	(0.090)	(0.147)	(0.107)	(0.091)	(0.089)	(0.105)	(0.178)
Level 4	0.00112	0.158	0.0444	−0.144	− 0.13	− 0.0642	− 0.15
	(0.094)	(0.153)	(0.112)	(0.095)	(0.093)	(0.109)	(0.186)
INSURANCE TYPE							
UEMBI	0	0	0	0	0	0	0
URMBI	−0.106^b^	− 0.0759	− 0.0343	− 0.017	− 0.0098	−0.0678	− 0.0223
	(0.047)	(0.078)	(0.057)	(0.048)	(0.047)	(0.055)	(0.094)
INSTITUTION TYPE							
Class III hospital	0	0	0	0	0	0	0
Class II hospital	−0.171^c^	− 0.382^c^	− 0.288^c^	− 0.166^c^	− 0.0478	0.0888	0.341^c^
	(0.051)	(0.084)	(0.061)	(0.052)	(0.051)	(0.060)	(0.102)
Class I hospital	−0.857^c^	−1.663^c^	−1.139^c^	− 0.843^c^	− 0.485^c^	− 0.328^a^	0.533
	(0.165)	(0.270)	(0.197)	(0.168)	(0.165)	(0.193)	(0.328)
Hospital for special							
General hospital	0	0	0	0	0	0	0
Special hospital	−0.274^c^	− 0.280^c^	− 0.284^c^	− 0.269^c^	− 0.227^c^	0.0795	0.117
	(0.062)	(0.102)	(0.074)	(0.063)	(0.062)	(0.073)	(0.124)
Clinic & Community	−0.174	0.563^a^	0.262	−0.268	−0.414^b^	− 0.242	− 0.428
	(0.206)	(0.337)	(0.247)	(0.209)	(0.206)	(0.241)	(0.410)
Pay-by-DRGs							
Yes	0	0	0	0	0	0	0
No	− 0.237^c^	−0.0938	− 0.0594	− 0.170^c^	− 0.219^c^	− 0.298^c^	−1.009^c^
	(0.054)	(0.088)	(0.065)	(0.055)	(0.054)	(0.063)	(0.107)
cons	8.456^c^	6.880^c^	7.376^c^	8.051^c^	8.247^c^	8.394^c^	9.303^c^
	(0.120)	(0.196)	(0.143)	(0.121)	(0.119)	(0.140)	(0.238)
*No.Obs*	2305	2305	2305	2305	2305	2305	2305
*R*^2^	0.36						

Note: as TTC was abnormal distribution, ln TTC was used to make the variable normalized

Standard errors in parentheses

^a^ Significant at the 90% level., ^b^ Significant at the 95% level, ^c^ Significant at the 99% level

**Table 12:** Results of ordinary least squares (OLS) and quantile regression for outpatients

Ln TTC	OLS	QR_10	QR_25	QR_50	QR_75	QR_90	QR_98
AGE	−0.003^c^	− 0.00004	0.005^c^	− 0.003^a^	− 0.003^c^	− 0.002^a^	− 0.008^c^
	(0.041)	(0.003)	(0.00171)	(0.002)	(0.001)	(0.001)	(0.002)
R _*reimbursement rate*_	0.789^c^	0.262	0.634^c^	0.972^c^	1.486^c^	1.716^c^	1.956^c^
	(0.066)	(0.142)	(0.0979)	(0.082)	(0.056)	(0.067)	(0.110)
OOP	0.0010^c^	0.0008^c^	0.0011^c^	0.0017^c^	0.0024^c^	0.0024^c^	0.0020^c^
	(0.00003)	(0.00004)	(0.00007)	(0.0002)	(0.0002)	(0.0002)	(0.0001)
GENDER							
Male	0	0	0	0	0	0	0
Female	−0.213^c^	−0.184^a^	−0.075	− 0.270^c^	− 0.196^c^	− 0.159^c^	− 0.338^c^
	(0.041)	(0.106)	(0.056)	(0.050)	(0.035)	(0.046)	(0.049)
AREA							
Eastern Area	0	0	0	0	0	0	0
Central Area	1.711^c^	2.312^c^	1.851^c^	1.320^c^	1.105^c^	0.673^c^	0.431^c^
	(0.133)	(0.345)	(0.182)	(0.162)	(0.112)	(0.150)	(0.159)
Western Area	−0.436^c^	−0.694^c^	−0.644^c^	−0.496^c^	−0.488^c^	0.0261	0.151
	(0.093)	(0.240)	(0.127)	(0.113)	(0.078)	(0.105)	(0.111)
CITY LEVEL							
Level 1	0	0	0	0	0	0	0
Level 2	−0.328^c^	−0.836^c^	−0.320^c^	−0.0901	− 0.0418	−0.0485	0.0788
	(0.054)	(0.141)	(0.074)	(0.066)	(0.046)	(0.061)	(0.065)
Level 3	−1.096^c^	−1.854^c^	−1.312^c^	−0.770^c^	−0.450^c^	− 0.254^c^	−0.253^c^
	(0.079)	(0.206)	(0.109)	(0.097)	(0.067)	(0.090)	(0.095)
Level 4	−0.507^c^	− 0.32	−0.434^c^	− 0.418^c^	−0.180^b^	− 0.409^c^	−0.495^c^
	(0.108)	(0.281)	(0.149)	(0.132)	(0.092)	(0.123)	(0.129)
INSURANCE TYPE							
UEMBI	0	0	0	0	0	0	0
URMBI	−0.131^a^	−0.851^c^	−0.722^c^	−0.175^b^	0.107^a^	0.267^c^	0.147^a^
	(0.070)	(0.182)	(0.096)	(0.086)	(0.060)	(0.080)	(0.084)
INSTITUTION TYPE							
Class III hospital	0	0	0	0	0	0	0
Class II hospital	0.138^a^	0.678^c^	0.635^c^	0.12	0.00232	0.0657	−0.230^c^
	(0.074)	(0.191)	(0.101)	(0.090)	(0.062)	(0.083)	(0.088)
Class I hospital	0.670^c^	0.844^c^	0.606^c^	0.425^c^	0.336^c^	0.478^c^	0.219^c^
	(0.063)	(0.163)	(0.086)	(0.077)	(0.053)	(0.071)	(0.075)
Hospital for special							
General hospital	0	0	0	0	0	0	0
Special hospital	0.102^b^	0.366^c^	0.432^c^	0.187^c^	−0.0111	−0.012	0.333^c^
	(0.051)	(0.133)	(0.070)	(0.062)	(0.043)	(0.058)	(0.061)
Clinic & Community	−0.960^c^	−0.311	− 0.840^c^	−0.487^c^	− 0.456^c^	−0.758^c^	− 0.895^c^
	(0.095)	(0.247)	(0.130)	(0.116)	(0.081)	(0.107)	(0.114)
Pay-by-DRGs							
Yes	0	0	0	0	0	0	0
No	−0.159^c^	0.0548	−0.149^a^	−0.309^c^	− 0.220^c^	−0.227^c^	− 0.266^c^
	(0.061)	(0.158)	(0.083)	(0.074)	(0.051)	(0.069)	(0.073)
cons	5.230^c^	3.515^c^	4.066^c^	5.197^c^	5.330^c^	5.467^c^	6.438^c^
	(0.088)	(0.204)	(0.104)	(0.153)	(0.102)	(0.143)	(0.217)
*No.Obs*	5442	5442	5442	5442	5442	5442	5442
*R*^2^	0.299						

Note: as TTC was abnormal distribution, ln TTC was used to make the variable normalized

Standard errors in parentheses

^a^ Significant at the 90% level., ^b^ Significant at the 95% level, ^c^ Significant at the 99% level

**Fig. 4:**
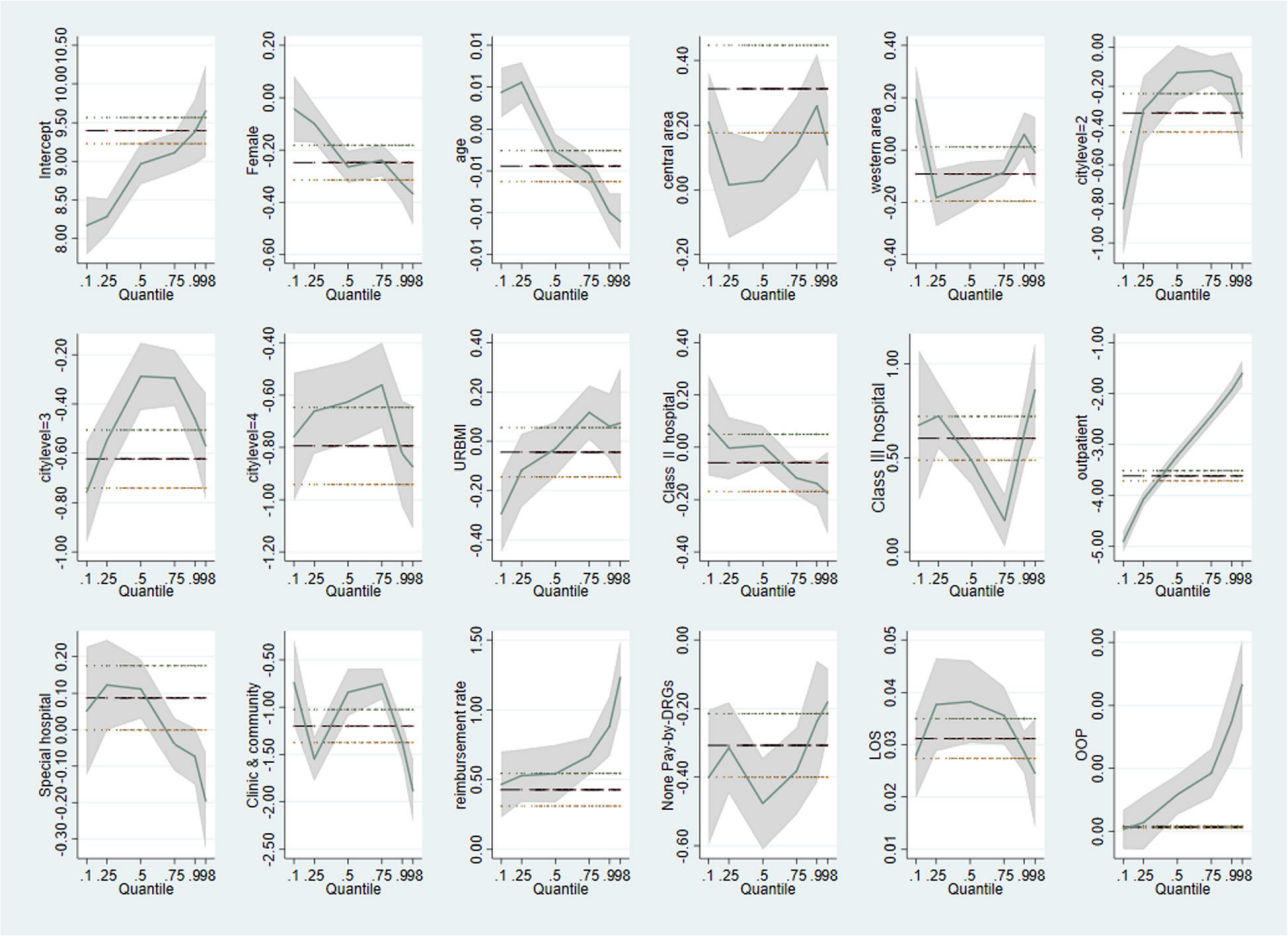
Quantile Regression versus OLS Regression. OLS: ordinary least squares

**Fig. 5:**
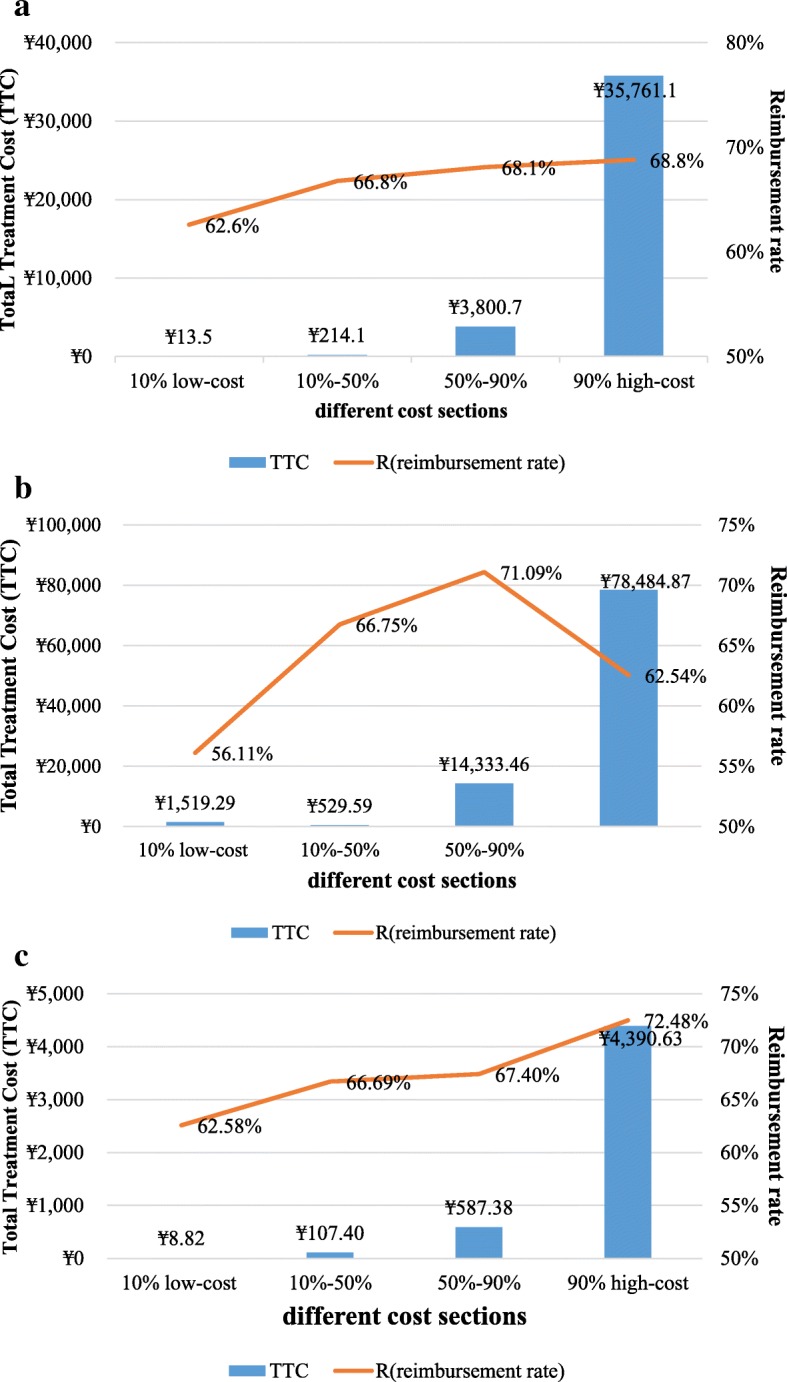
Insurance reimbursement and total treatment cost of different cost sections. **a** overall insurance reimbursement and total treatment cost of different cost sections. **b** insurance reimbursement and total treatment cost of different cost sections for inpatients. **c** insurance reimbursement and total treatment cost of different cost sections for outpatients

In contrast, quantile regression, showed a different effect of the factors on the TTC at different quantiles. The effect of areas, insurance type and kinds of hospitals were not exactly the same as in OLS. Compared to patients with UEMBI, URMBI had a significant negative impact on TTC at the 10th percentile, which declined the TTC by 30% at the 5% significance level; while, at the 75th and 90th percentiles, URMBI increased the TTC by 12% at the 1% significance level, and by 6% at the 5% significance level (Tables 10, 11 and 12, Fig. 4). Both OLS and quantile regression showed that TTC was significantly associated with reimbursement rate. In general, *R*_*reimbursement rate*_ on the 90% high-cost group was greater than that on the 10% low-cost group (Fig. 5a). The effects of reimbursement on TTC were small at the lower quantiles (below the 50th percentiles) but more pronounced at the upper quantiles, which could increase TTC by 90% at the 90th percentile (Table 10, Fig. 4). *R*_*reimbursement rate*_ was highly associated with the TTC than other factors.

The effects of influencing factors were quite different between inpatients and outpatients with the quantile regression. Compared with inpatients, gender (*P* < 0.01), areas (P < 0.01), city levels (P < 0.01), hospital types ((*P* < 0.05) were significantly associated with TTC for outpatients (Tables 11 and 12). *R*_*reimbursement rate*_ was different in highly cost quantiles (upper quantiles, that above the 50th percentiles) for inpatients and outpatients. For inpatients, the quantile regression results were different from the general trend (Fig. 5).

## Discussion

Rare disease is not rare in China, as it has the largest population in the world [5, 29]. Lack of effective treatment for and misdiagnoses, missed diagnoses, and incurability of rare diseases result in both physical and mental stress for years [51]. Medicine costs of the 7,747 records from 29 provinces accounted for over half of the TTC, and 60% of insurance payment type was paid for the service. Different subgroups showed the significant differences in terms of medical cost. Citizens with URBMI had a higher disease burden than did those with UEBMI. The institution classification (by function and level), city level, and reimbursement rate were associated with medical cost.

Most of the rare diseases are genetic diseases, incurable, and could affect the patients throughout their life. Without specific diagnoses and adequate treatment, patients with rare disease experience considerable disease burden [5, 7]. Affordable treatment is an urgent need [9]. During the study period, patients with rare diseases consumed a total of ¥40.13 million (£4.48 million). Further, 10.8% of the patients could have suffered from poverty, as their direct medical expenses had reached the value of CHE.

Accessibility of effective treatment is also one of the most important issues, many minority groups like people with rare diseases still face challenges in receiving equal healthcare access [13]. Currently, Class III hospitals hold the main position with respect to the diagnoses and treatment for rare diseases, as these hospitals are at the top-tier health service provider in China [52]. It was seen that 72.6% of patients with rare diseases received their medical treatment in Class III hospitals. According to another report, China’s top-tier hospitals are concentrated in developed areas, like Beijing, Shanghai, located in eastern area [52]. With higher service level and better technology, the health service utilization of patients with rare diseases is better in developed regions than in developing regions [5]. This is also why 73.8% of the records coming from Eastern China. Obviously, high-quality health resources, such as advanced equipment, and high-qualified skills contribute to a more effective treatment. This might explain the differences of medical expenses among different service institutions, different areas, and why Class III hospital, Eastern area, or developed cities (level 1 city), had a significantly lower cost.

Patients with rare diseases experience a considerable financial burden compared to the national average level [9, 15, 53]. It is worth noting that this would undoubtedly increase the burden on basic health insurance. Some provinces and cities, such as Ningxia province, Shanghai, and Qingdao, have included common rare diseases into the local insurance policy [13, 54–56]. However, a national special issue for rare diseases could not be developed until June, 2018 [38, 54, 55]. The absence of policy could lead to inadequate security and support for this group. During the study period, there were still 11.9% cases that paid by individual only. Meanwhile, healthcare insurance system only covers the service in hospitals, and some patients could choose to buy pharmaceuticals outside the hospital, and medical insurance data could not equal to total cost. The diseases burden might be heavier. More specifically, patients with URBMI had higher medical costs than did those with UEBMI. As UBMI applicants are residents such as juveniles and elder people, without jobs and a stable income, the high TTC might increase the disease burden for not only patients but their families.

R _*reimbursement rate*_ and OOP were strong positive influencing factors for patients with rare disease. The effect of reimbursement on the 90% high-cost group was greater than that on the 10% low-cost group for both inpatient and outpatient services. This indicates that the basic insurance has an effect on supporting patients with rare diseases that entail high expense. However, the coverage level was limited [57]. In this study, it was shown that for patients with rare diseases, OOP accounted for 32.9% of the TTC, which was 0.9% higher than the national average level. Furthermore, the quantile analysis showed that, although the factors were almost similar to different medical cost percentiles, the affect is significantly different between the high-cost and low-cost segments. The impact of urban residence on medical costs and medical utilization is equally evident, especially for the low and high cost segments. It confirms the stimulating effect of social-economic development level on medical service ability and medical service demand release.

At the same time, different diseases involve different examinations and therapies, which may affect the reimbursement rate, and this is the same for rare diseases. With respect to the coverage target, the basic healthcare insurance aimed to provide a financial guarantee for basic healthcare demands. It was, however, difficult to balance the basic health insurance coverage for patients with both common and rare diseases. More progress is needed [51]. The huge disparity between medical cost and security level of basic health insurance for different rare diseases in this study indicates the urgent need for implementing the Critical Illness Insurance. Pay-by-DRGs had been added to the multi-tiered and comprehensive medical insurance payment system. Nowadays, DRGs-PPS is an observed tendency in the payment reform of health care insurance [17, 18]. Different areas had their local reform plan with the usage of pay-by-DRGs (e.g. Shandong, Zhejiang, Beijing, Guangdong), and had their specific DRGs [13, 54–56]. Although there is not a national insurance disease list for rare disease in China, and DRG-PPS doesn’t cover all of these rare diseases. Lack of diagnosis experience, medicine and treatment, it makes rare disease more likely be treated as other common disease had same complications or symptoms which had been added to the list of DRGs. That could explain why there were usage of pay-by-DRGs in these rare diseases (e.g. Qingdao City in Shandong Province, Guangzhou City in Guangdong Province). Meanwhile, the cover rate had been higher year by year, which could indicate the tendency of DRG-PPS. Furthermore, the study found that the implementation of paid by DRGs makes it possible to control TTC and reduce the disease burden of patients. This might also be considered as an effective way in medical cost control for the governors.

Lastly, lack of experience in and knowledge of rare diseases result in repeated testing and ineffective treatments and thereby an increase in medical cost. Due to the low accuracy of diagnosis of rare disease, nearly 65% of patients with rare diseases have been misdiagnosed [7]. It has been found that less than 7% of doctors are specialized in rare disease, while almost 30% of doctors have no experience in treating rare diseases [7, 9]. Therefore, few or no records of rare diseases in a particular regions do not indicate the absence of rare diseases but the possibility of misdiagnoses and or a missed diagnosis [23]. It is gratifying that a national medical treatment network has been initially established to further improve rare disease care [58]. However, there are a long journey in the protection of rare disease patients, as reported only 121 kinds of disease and less than 30 orphan drugs had been covered by basic health insurance [59].

### Limitations

As a data-based evidence from Mainland China, this study is the first try to present the situation of health security of patients with rare diseases. Six of the eight certain rare disease were included in the National Rare disease List (version 2018) 2 year after our research, which could make our study as a typical retrospective study for the evaluation of security level of healthcare insurance for patients with rare diseases to some extent. However, there were some limitations of this study. Firstly, NCMS and UBMI have covered over 95% of the citizens of China. Before the reform of the central administration of 2018, UBMI was operated by the Ministry of Human Resources and Social Security, while NCMS was operated by National Health Commission of China. The database from CHIRA that we used in this study was supervised by the Ministry of Human Resources and Social Security, which only contained the insurance information regarding UBMI. Previous studies have reported significant disparities with respect to healthcare utility and health service quality between urban and rural areas or developed areas and developing areas. Therefore, to some extent, the findings of our study do not accurately represent the situation of patients with rare diseases covered by NCMS. Secondly, considering the morbidity rate of rare disease, the population of each individual disease was small, we took patients with the 8 rare diseases as a whole group to study with the health security situation for rare disease patients in China for the first step. And for a better explanation effect of multivariate regression analysis, data were not analysed by considering each disease separately. Thirdly, this database is only a daily supervision system for healthcare utility data in hospitals, and some patients could choose to buy pharmaceuticals outside the hospital. It was reported that drugstores shared about 20% of medicine market in China [60], which meant that the current cost data could not equal to the total cost, but the results could still reveal diseases burden in a large part. Meanwhile, according to the National health statistical rule in China, each visit of patients was estimated including multiple visits, and the study focused on the healthcare utility of each visit record instead of patients.

## Conclusion

Healthcare insurance is an effective safeguard for patients with rare diseases; however, affordable and accessible treatment is still lacking for rare disease patients. The society and government should build a healthcare security system from equality, sustainable and inclusiveness perspective. Undoubtedly, there is still a need to further improve the diagnostic and treatment technology, expertise among healthcare providers regarding rare diseases, as well as the health security level. Therefore, first, the treatment for rare disease needs to be improved, especially in developing areas. Second, the universal health coverage system needs to be more accurate and targeted, such as by improving the reimbursement level, with attention to control medical cost; further integration of basic medical insurance to ensure the equity of health security for different groups of people; distinguishing the risk of poverty among different, and improving the role of supplementary insurance in the universal health coverage system.

## Additional file

Additional file 1: **Appendix I** a) Basic status of different areas of China. b) Per capita disposable income by province in 2014-2016. **Appendix II** a) rare disease for the selection. b) 8 rare diseases with high morbidity and specific diagnostic criteria. **Appendix III** Basic introduction of UMI and RMI. **Appendix IV** National total treatment cost for inpatients and outpatients in 2014 to 2015. (DOCX 39 kb)

## Data Availability

Data may be available from the corresponding author after any reasonable requests or get from CHIRA.

## Abbreviations

CHE: Catastrophic health expenditure
CHIRA: The China Healthcare Insurance Research Association
DRGs: Diagnostic related groups
DRGs-PPS: Diagnostic related groups-prospective payment system
LOS: Length of stay
NCMS: The New Cooperative Medical Scheme
NHC: The National Health Commission of China
OLS: Ordinary Least Squares
OOP: Out-of-pocket costs
TTC: The total treatment cost
UBMI: Urban Basic Medical Insurance
UEBMI: The Urban Employees’ Medical Insurance
URBMI: The Urban Residents Medical Insurance
WHO: World Health Organization
