# Supplementary material for: Health service security of patients with 8 certain rare diseases: evidence from China’s national system for health service utilization of patients with healthcare insurance
Source: Orphanet J Rare Dis. 2019 Aug 20;14:204. doi: 10.1186/s13023-019-1165-7 (PMC6700821; doi:10.1186/s13023-019-1165-7)
Supplement: Supplementary file 1 — Appendix I a) Basic status of different areas of China. b) Per capita disposable income by province in 2014-2016. Appendix II a) rare disease for the selection. b) 8 rare diseases with high morbidity and specific diagnostic criteria. Appendix III Basic introduction of UMI and RMI. Appendix IV National total treatment cost for inpatients and outpatients in 2014 to 2015. (DOCX 39 kb) [file 13023_2019_1165_MOESM1_ESM.docx]

Appendix Ⅰ **a) Basic status of different areas of China**

|  | | Eastern China | Central China | Western China |
| --- | --- | --- | --- | --- |
| Provinces | | 11 provinces/ municipalities  Beijing, Tianjin, Hebei, Liaoning, Shanghai, Jiangsu, Zhejiang, Fujian, Shandong, Guangdong and Hainan | 8 provinces  Shanxi, Jilin, Heilongjiang, Anhui, Jiangxi, Henan, Hubei and Hunan | 11 provinces  Chongqing, Sichuan, Guizhou, Yunnan, Tibet, Shaanxi, Gansu, Inner Mongolia, Ningxia, Qinghai and Xinjiang |
| Populations  (*10^6^) | 2014 | 562.08 | 426.71 | 366.37 |
|  | 2015 | 565.60 | 428.47 | 367.39 |
|  | 2016 | 573.29 | 430.54 | 375.07 |
| hospitals | 2014 | 9500 | 7309 | 7900 |
|  | 2015 | 9937 | 8663 | 8371 |
|  | 2016 | 10580 | 8051 | 8950 |
| Outpatient  Service  (*10^6^) | 2014 | 1546.82 | 600.01 | 594.93 |
|  | 2015 | 1662.85 | 660.27 | 648.95 |
|  | 2016 | 1708.50 | 690.00 | 685.14 |
| inpatient  Service  (*10^6^) | 2014 | 72.65 | 60.38 | 59.13 |
|  | 2015 | 77.87 | 64.62 | 61.93 |
|  | 2016 | 80.00 | 67.06 | 63.48 |

**Appendix Ⅱ: a)** **rare disease for the selection**

| **Disease Name*** | **Step 1*^a^*** | **Step 2*^b^*** | **Step 3*^c^*** |
| --- | --- | --- | --- |
| Acute promyelocytic leukemia | √ | √ | √ |
| Albinism | √ |  |  |
| Amyotrophic lateral sclerosis/Motor neuron disease | √ | √ | √ |
| Freeman-Sheldon syndrome | √ |  |  |
| Growth hormone deficiency | √ | √ | √ |
| Haemophilia/hemophilia | √ | √ | √ |
| Huntington disease | √ | √ |  |
| Kallmann syndrome | √ | √ |  |
| Langerhans cell histiocytosis | √ |  |  |
| Lymphangioleiomyomatosis | √ |  |  |
| Mucopolysaccharidosis | √ | √ | √ |
| Multiple sclerosis | √ | √ | √ |
| Myasthenia gravis | √ | √ | √ |
| Niemann-Pick disease | √ |  |  |
| Paroxysmal nocturnal hemoglobinuria | √ |  |  |
| Phenylketonuria | √ | √ | √ |
| Primary pulmonary hypertension | √ |  |  |
| Tuberous sclerosis | √ |  |  |
| Turner syndrome | √ |  |  |
| Tyrosinemia | √ |  |  |

Note: * diseases are reported by media had high morbidity in China The references are as follows: [1] *South metropolis daily. 24 common rare diseases in China[R]* [*http://epaper.oeeee.com/epaper/A/html/2014-09/03/content_3307352.htm?div=0*](http://epaper.oeeee.com/epaper/A/html/2014-09/03/content_3307352.htm?div=0) [2] *Rare disease in China. Search for rare disease[EB/OL].* [*http://www.hanjianbing.org/disease!index*](http://www.hanjianbing.org/disease!index) [3] Chinese Organization for Rare Disorders. *Reference list of rare diseases in China [EB/OL].* <http://www.hanjianbing.org/upload/file/20180809/1533804248364053608.pdf>

a) 20 certain rare diseases, with a high morbidity rate and those familiar to Chinese public were selected with literature review to choose rare diseases and focus group discussion.

b) ten rare diseases were selected randomly from the 20 options.

c) two rare diseases with ultra-low incident rate (patient less than 10 in three years) were excluded from the database.

**Appendix Ⅱ: b)** 8 rare diseases with high morbidity and specific diagnostic criteria

| **Disease Name** | **Abbreviation** | **ICD-10 Codes** | **Clinical Information** | **Incidence/Morbidity** |
| --- | --- | --- | --- | --- |
| amyotrophic lateral sclerosis**  Motor neuron disease | ALS/MND  Lou Gehrig's disease | G12.2 | is a progressive disease that affects motor neurons, which are specialized nerve cells that control muscle movement. | 1～3/100,000 |
| acute promyelocytic leukemia | APL | C92.4 | is an aggressive type of acute myeloid leukemia in which there are too many immature blood-forming cells (promyelocytes) in the blood and bone marrow. | 0.23/100,000 |
| growth hormone deficiency | GHD | E23.0 | is a rare disorder characterized by the inadequate secretion of growth hormone (GH) from the anterior pituitary gland, a small gland located at the base of the brain that is responsible for the production of several hormones. | 10~25/100,000 ^a^ |
| Haemophilia/hemophilia** |  | D66-D68 | any of several X-linked genetic disorders, symptomatic chiefly in males, in which excessive bleeding occurs owing to the absence or abnormality of a clotting factor in the blood.  The symptoms of haemophilia can be mild to severe, depending on the level of clotting factors you have. The main symptom is bleeding that doesn't stop, also called prolonged bleeding. | 2.73/100,000 |
| Myasthenia gravis/  Generalized Myasthenia gravis ** | MG | G70.0 | is a disorder that causes weakness of the skeletal muscles, which are muscles that the body uses for movement. Congenital myotonia syndrome（Non-Dystrophic Myotonia, NMD）and General Myathenic Gravis | 20/100,000 |
| mucopolysaccharidosis** | MPS | E76.0  E76.1  E76.3 | a group of metabolic disorders caused by the absence or malfunctioning of lysosomal enzymes needed to break down molecules called glycosaminoglycans. | 0.36~1.33/100,000 ^b^ |
| multiple sclerosis** | MS | G35 | is a condition characterized by areas of damage (lesions) on the brain and spinal cord. | 0-5/100,000 |
| phenylketonuria** | PKU | E70.0 | A genetic disorder in which the body lacks the enzyme necessary to metabolize protein.  PKU is an inherited disorder that increases the levels of a substance called phenylalanine in the blood | 8.5/100,000 |

Note: ** diseases recorded in the national list of rare diseases of China (first version).

a) the incidence of GHD has not been reported in China, this date is related to the frequency of isolated GHD in USA.

b) the incidence of MPS has not been reported in China, this date is related to the incidence of USA, Northern Ireland and Netherlands.

**Appendix Ⅲ: Basic introduction of urban medical insurance (UMI) and rural medical insurance (RMI)**

|  | **UEMI** | **URMI** | **RMI** |
| --- | --- | --- | --- |
| **Overseeing ministry** | Ministry of human resource and social security, MoHRSS | Ministry of human resource and social security, MoHRSS | National health commission, NHC |
| **Level of pooling** | Prefecture/municipality | Prefecture/municipality | County |
| **No. of risk pools (approx.)** | 330 | 330 | 2,600 |
| **Enrolment rate(%)** | >95 ^a^ | >95 ^a^ | 98.9^b^ |
| **Number of enrollees, million** | 377 million | 288 million | 670 million |
| **Unit of enrollment** | individual | individual | household |
| **Source of premium** | Employer and individual | Government  and individual without jobs | Government and individual |
| **Benefits package** | in- and outpatient care  critical and chronic outpatient care | in- and outpatient care  critical and chronic outpatient care | in- and outpatient care  critical and chronic outpatient care |
| **Reimbursement (yuan)** | 753.2 billion | 178.1 billion | 289.04 billion |
| **Mandated reimbursement rate(%)** | | | |
| -inpatient care | >80% | >70% | >75% |
| -outpatient care | no | no | no |
| **Effective reimbursement rate(%)**^c^ | | | |
| -inpatient care | 72.0 | 54.4 | 58.5 |
| -outpatient care | >50 | 50 | 50 |

*Sources: China Health Statistics Yearbook 2015* (NHC). *Summary of National Social Insurance in 2015* [in Chinese] (MoHRSS). *Statistical Bulletin of the Development of Human Resources and Social Security, 2015* [in Chinese] (MoHRSS). *The New Rural Cooperative Medical Scheme Statistical Manual, 2013* [in Chinese] (NHC). *Policy of the* *New Rural Cooperative Medical Scheme* (2012-2015) [in Chinese] (NHC). *Policy of healthcare insurance* (2012-2015) [in Chinese] (MoHRSS). “[1]Green book of health reform and development: Annual report 2016, special issue for reform and development in healthcare insurance system [in Chinese] (PQ Fang, et al.).

*Note:* a. reported in *The 12th five-year report: achievements in employment and social security* (MoHRSS,2015)

b. reported in *China Health Statistics Yearbook 2015* (NHC,2015)

c. reported in *Green book of health reform and development: Annual report 2016*

**Appendix Ⅳ: National total treatment cost for inpatients and outpatients in 2014 to 2015**

|  | **2014** | **2015** | **2016** | **Average** |
| --- | --- | --- | --- | --- |
| **Total cost per inpatient service (￥)** | 7,442.3 | 7,832.3 | 8,268.1 | 7,866.34 |
| **Total cost per visit of outpatient TTC (￥)** | 206.4 | 220.0 | 233.9 | 220.63 |
| **Inpatient service (*10^6^)** | 140.07 | 153.75 | 160.87 | 151.56 |
| **outpatient service (*10^9^)** | 2.74 | 2.97 | 3.08 | 2.93 |

*Source：China Health Statistics Yearbook 2014-2016 (NHC).*
